# RNA-seq Transcriptome Analysis in Ovarian Tissue of Pelibuey Breed to Explore the Regulation of Prolificacy

**DOI:** 10.3390/genes10050358

**Published:** 2019-05-10

**Authors:** Wilber Hernández-Montiel, Reyna Cristina Collí-Dula, Julio Porfirio Ramón-Ugalde, Mario Alberto Martínez-Núñez, Roberto Zamora-Bustillos

**Affiliations:** 1 División de Estudios de Posgrado e Investigación, TecNM/Instituto Tecnológico de Conkal, Av. Tecnológico S/N, Conkal, Yucatán 97345, Mexico; wilber.hernandez@itconkal.edu.mx (W.H.-M.); julio.ramon@itconkal.edu.mx (J.P.R.-U.); 2 Departamento de Recursos del Mar, Cinvestav Unidad Mérida, Mérida, Yucatán 97310, Mexico; rcolli.dula@cinvestav.mx; 3 UMDI-Sisal, Facultad de Ciencias, Universidad Nacional Autónoma de México, Sierra Papacal-Chuburna Km 5, Mérida, Yucatán 97302, Mexico

**Keywords:** genes, RNA-seq, reproductive processes, differentially expressed gene

## Abstract

The Pelibuey sheep (*Ovis aries*) is an indigenous breed distributed in the tropical regions of Mexico. The prolificacy of this sheep is on average from 1 to 1.5 lambs, being an important breeding characteristic that owners seek to increase with the purpose of economic improvements. New-generation RNA sequencing technology has been used to identify the genes that are expressed in the ovarian tissue of sheep that have two or more lambs per parturition, as well as to elucidate the metabolic pathways that are affected by the expression of these genes, with the purpose of better understanding the prolificacy in the sheep. In the present study, the transcriptional expression of multiparous and uniparous sheep was compared using RNA sequencing. Multiparous (M group) and uniparous (U group) sheep that had a genealogical record for three generations (M, n = 5 and U, n = 5) were selected. RNA was extracted from ovarian tissue and subsequently used to prepare the libraries that were sequenced using the Illumina NextSeq500 platform. A total of 31,575 genes were detected from the transcriptomic analysis of which 4908 were significantly expressed (*p*-value ≤ 0.001) in the ovary of sheep. Subsequently, a second filter was carried out to evaluate the false discovery rate (FDR) and select those genes with *p*-values ≤ 0.05 and values of expression ≥ 1 (log2), obtaining 354 differential expressed genes (DEG): 120 genes up-regulated and 234 genes down-regulated in the group M with respect to the group U. Through Gene Ontology (GO) and metabolic analysis, we obtained information on the function of differentially expressed genes, and its importance in the reproduction of multiparous sheep. This result suggest that genes identified in the present study participate in the development of the final stages of follicles.

## 1. Introduction

Reproductive efficiency is considered one of the most important factors affecting the sheep meat industry. It is directly related to traits such as ovulation rate and litter size, which are genetically affected by causative mutations in some minor and major genes [1] and their interactions with the environment. The litter size improvement is one of the most important objectives for sheep breeders as it greatly increases production efficiency and economic gains [2], and therefore its analysis with molecular tools is of great importance for the profitability of sheep production. The Pelibuey sheep (*Ovis aries*) is an indigenous breed, distributed in the tropical regions of Mexico with adaptability to climatic variations, maternal capacity with a low reproductive seasonality that allows productivity throughout the year and an average litter size from 1–1.5 [3,4]. Therefore, the study of its reproductive efficiency through the analysis of the genetic expression associated with prolificacy will allow for better profitability of its breeding.

The ewes, as well as other species, present irregularities in the cycle of follicular development, due to the environmental interaction and the regulation of intraovarian expression genes, which occurs in the folliculogenesis process [5]. In recent years, a group of genes with different mutations (SNPs) has been identified and is associated with prolificacy in different wool sheep breeds. Within this group is the family of the transforming growth factor β (TGF-β) genes, where the SNPs result in an increased ovulation rate (OR) in sheep [2,5]. The bone morphogenetic protein receptor 1B (*BMPR1B*) gene, and their SNP identified as *FecB^B^*, are associated with hyperprolific phenotypes of Booroola ewes [6]. The bone morphogenetic protein 15 (*BMP15*) gene, has been reported with eight SNPs associated with prolificacy: *FecX^I^*, *FecX^H^*, *FecX^B^*, *FecX^G^*, *FecX^L^*, *FecX^O^*, *FecX^Gr^*, and *FecX^2W^* [5]. So far, 11 SNPs have been identified for the Growth Differentiating Factor 9 (GDF9) gene, labeled *G1–G8* [7]; *FecG^T^*, *FecG^E^* and *FecG^H^* [5]. However, the mutations reported in the TGF-β genes are not present in all sheep-producing meat breeds, including hair sheep breeds from African origin, such as D’man [8], Afshari, Ghezel, Lori-Bakhtyari and Afshari breeds from Iran [9], Grivette from France [10] and Black Belly from Mexico [11]. The absence of these mutations in these sheep breeds, could indicate that there are not unique genes that intervene in litter size [12], and there may be other molecular mechanisms involved in the prolificacy of sheep.

Currently, next-generation sequencing (NGS) tools are used for the identification of differentially expressed genes (DEGs) in the biological processes, and particularly in recent years, have been used for the identification of genes differentially expressed in sheep ovarian tissue with high economic value [13,14]. Some examples of the use of RNA-seq technology are those carried out with Qira Black and Hetian sheep, which are characterized by high-fecundity and low-fecundity, respectively. Their messenger RNA (mRNA) expression profiles in the ovaries were compared and a total of 1252 DEGs were identified. Some genes were associated with the processes of foliculogenesis and prolificacy traits, such as, *PRLR*, *PGR*, *ESR2*, *CYP19A1*, *CYP11A1*, *HSD17B12* and *INHBA* [13]. Similarly, analysis of the expression profile of granulosa cells (GC) from Hu sheep, found that 170 genes were significantly differentially expressed; among the up-regulated genes are *ADCY7*, *CD44*, *PTGS2*, *RAC2*, *IL1A*, *IL1B*, *TNF*, *CYP2S1*, and *PRL;* while among down-regulate genes are *CYP19A1*, *IGFBP1* and *THBS2*, which are related to the functional category of follicular development according to the Kyoto Encyclopedia of Genes and Genomes (KEGG) database [14]. The aim of the present study was to identify differentially expressed genes through the transcriptomic analysis of the ovarian tissue of multiparous and uniparous Pelibuey ewes, as well as the metabolic pathways that are significantly expressed in them using RNA-seq methodology, to better understand reproductive biology and provide a molecular basis for new methods of genetic improvement for this important breed.

## 2. Materials and Methods

Two groups of Pelibuey (n = 10) ewes were selected based on the criteria of number of lambs per partum, and with records of three consecutive births. All sheep in the study were from a commercial sheep farm “El Cortijo”, located in Campeche, Mexico (19°43′48.92″ N and 90°05′11.58″ W). The animals were transported and kept in the facilities of CESyRO (Center of Selection and Ovine Reproduction) of Technological Institute of Conkal, Yucatan, México. The conditioning of the animals was supervised by the ethics committee of the institute following the official Mexican standard (NOM-033-SAG/ZOO-2014).

### 2.1. Synchronize Estrus and Sample Preparation

The 10 experimental sheep were divided into two groups of five animals, referred to as Group M and Group U. Group M corresponds to multiparous sheep with two lambs per birth (M, n = 5), and group U corresponds to uniparous sheep with a single lamb at birth (U, n = 5). The animals of both groups were fed the same diet of Taiwan grass (*Pennisetum purpureum*) and 250 g/ewe/d of a commercial supplement with 16% crude protein, mineral lick and water *ad libitum*. The estrous cycle of the sheep was synchronized using intravaginal sponges of fluorogestone acetate (40 mg; Chonogest^®^, Intervet, Santiago Tianguistenco, México) for 14 days, followed by intramuscular application of 1000 UI of gonadotropina (Folligon^®^). Ovarian tissue samples were obtained by biopsy using a left unilateral ovariectomy, immediately stored at −80 °C until RNA extraction.

### 2.2. RNA Extraction

For RNA extraction, 50 mg of tissue was taken from each sample using the Trizol Reagent according to the manufacturer’s protocols (Invitrogen™, Carlsbad, CA, USA). The RNA was treated with Turbo DNA-free (Ambion, Austin, TX, USA). The quantity and quality of RNA were measured using the Agilent 2100 BioAnalyzer system (Agilent Technologies, Santa Clara, CA, USA) with the RNA 6000 Nanochip. RNA Integrity values (RIN) were >7.0 for all samples used in the analysis.

### 2.3. Library Preparation and Sequencing

Samples were processed following the manufacturer’s protocol for NEBNext Ultra™ RNA Library Prep Kit for Illumina (New England Biolabs (NEB), Ipswich, MA, USA) in conjunction with the NEBNext Poly (A) mRNA Magnetic Isolation Module and the NEBNext Multiplex Oligos for Illumina (Index Primers Set 1) NEB. This step was followed by first-strand cDNA synthesis using reverse transcriptase and oligo-dT primers. Synthesis of ds-cDNA was done using the 2nd strand master mix provided in the kit, followed by end-repair and dA-tailing. This was done to add universal Illumina adaptors to the samples. Finally, the library was enriched (and barcoded) by 6-9 cycles of amplification, and purefied by Agencourt AMPure beads (Beckman Coulter, Brea, CA, USA). The library size and mass were assessed by analysis in the Agilent 2100 BioAnalyzer (Tape Station). Briefly, 10 μL of library (4 nM) was mixed with 10 μL 0.1 N NaOH for 5 mins, then the library was diluted to 20 pM in HT1 buffer and sequenced using the reagents provided in the Illumina 150-cycle kit and following the manufacturer’s protocol for high output NextSeq500 sequencing.

### 2.4. Data Analysis and Differential Expression Analysis

The raw data obtained from the sequencing by RNA-seq were first filtered to remove adaptors, as well as low-quality reads, using the NGS QC Toolkit v2.3.3 [15] software, and its program IlluQC.pl for Ilumina data using default parameters. Subsequently, to carry out the transcriptomic study of the filtered sequences, an analysis of the RNA reads was carried out with the strategy of using a reference genome. The TopHat v2.1.1 program was used [16] to assemble the reads, and the genome of the sheep *O. aries* v4.1 was used as reference genome to map the filtered reads and downloaded from NCBI genomes database (https://www.ncbi.nlm.nih.gov/genome?term=Ovis%20aries). The expression level of the genes in each sample were estimated and normalized as RPKM (Reads Per Kilobase Million Mapped Reads) [17], which is a normalized measure of read density that allows transcript levels to be compared both within and between samples. Cuffdiff v2.2.1 software [16], was used to identify the DEGs between the two groups, and a *p*-value was assigned to each gene to evaluate its statistical significance. Then we determine the false discovery rate (FDR) of the test to account for Type I errors. Multiple-testing corrections were performed using the Benjamini and Hochberg step-up false-discovery rate (FDR)-controlling procedure to calculated adjusted *p*-values. Genes with an adjusted *p*-value ≤ 0.05 and an expression |log2 ratio| ≥ 1 were identified as DEGs.

### 2.5. Analysis of Gene Ontology Category and Kyoto Encyclopedia of Genes and Genomes (KEGG) Pathway

To obtain information on the function of differentially expressed genes, and its importance in the reproduction of multiparous sheep, an enrichment analysis was performed for up-regulated and down-regulated genes, using the online platform DAVID [18]. The list of selected DEGs obtained from the transcriptomic analysis made with TopHa was sent to DAVID database to obtained their biological functions using Gene Ontology (GO) terms [19], which analyzes three functional categories: biological process (BP), cellular component (CC), and molecular function (MF). To obtain an overview of the DEGs and the different metabolic pathways in which they participate, the KEGG database was used [20]. To graph the GO enrichment analysis obtained from the DAVID database, the WEGO v2.0 program was used [21].

### 2.6. Validation of RNA-Seq Data by Quantitative Real-Time Polymerase Chain Reaction

The same tissues used for RNA-seq were used for quantitative real-time polymerase chain reaction (qRT-PCR) analysis. The RNA was treated with the DNase I enzyme (TURBO DNA-free™, Ambion^®^). For cDNA synthesis, DNase-treated RNA was used with the RevertAid First Strand^®^ kit (Thermo Scientific^®^, Lithuania). The real-time PCR were performed in a Thermal Cycler (iCycler IQ5; Bio-Rad, Hercules, CA, USA) with specific primers designed (Table 1). Each analysis was performed in triplicate with the Maximum SYBR^®^ Green/Fluorescein qPCR kit (Thermo Scientific^®^) according to the manufacturer’s instructions. The reaction of each sample resulted in a final volume of 25 µL, constituting of 12.5 μL of SYBR Green/Fluorescein (2X), 0.3 μM of each oligonucleotide and 2000 ng of RNA. The thermal protocol was 95 °C for 30 s and 35 cycles of 64 °C for 1 min and 72 °C for 30 s. The data were analyzed with iQ5 optical system software v.2 (Bio-Rad). Finally, the method of 2^−ΔΔCT^ by Livak et al. [22], was used to calculate fold changes. The significance of the expression levels between the control group (β-actin) and the treatments (*CA5A* and *FEM1B*) were determined for analysis using the Mann–Whitney U-test and Kruskal–Wallis test. Statistical analyses were performed using Sigma Plot (SYSTAT, Chicago, IL, USA) and figures were plotted using Prism 5.0 (GraphPad Software Inc., San Diego, CA, USA).

## 3. Results

**Figure 1:**
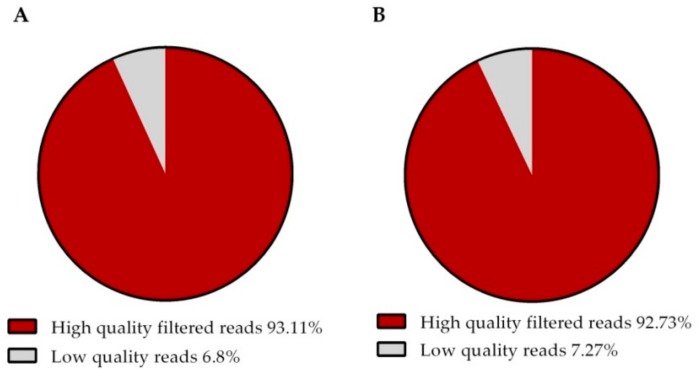
Composition total raw reads of the quality control (QC) that represents the percentage of readings of the groups: (**A**) Summary of quality check and filtering in multiparous sheep; (**B**) Summary of quality check and filtering in uniparous sheep.

Using the Illumina NextSeq500 sequencing platform, a total of 64,998,983 paired-end (PE) reads were obtained for group M, while 61,941,256 PE reads were obtained for group U. The quality control resulted in 60,528,631 (93.11%) reads for group M and 57,442,547 (92.73%) reads for group U (Table 2) and their results indicate that they were of high quality (Figure 1).

### 3.1. Identification of Differentially Expressed Genes

The transcriptomic analysis of the two sheep groups showed a total of 31,575 genes detected using the reference genome of *O. aries* v4.1. After selecting those genes with a *p*-value ≤ 0.001, were obtained 4908 genes for group M, of these 2706 genes were up-regulated, and 2202 genes were down-regulated. In a subsequent step, a Benjamini and Hochberg test to control false discovery rate (FDR) was done. The genes with *p*-value ≤ 0.05 and fold change ratio (log2) ≥ |1| were identified as DEGs, obtaining 120 genes up-regulated and 234 genes down-regulated in the group M (Table S1). The top 10 differentially expressed genes involved in the ovary development process in ewes with two lambs at birth were: *FGF18, CA5A, SVIP, CXCL14* and *FST* up-regulated; while the down-regulated were *RENT, FEM1B, MMP9, CYP2S1* and *HOXA9* (Table 3). The set of most relevant genes identified in the present analysis and that has been reported in other studies were *IL1A, CYP2S1, BMP15*, and *INHIB* [13,22], involved in reproductive processes as regulatory factors in ovarian development and in the ovulation process in female mammals [23,24].

### 3.2. Gene Ontology and KEGG Analysis

**Figure 2:**
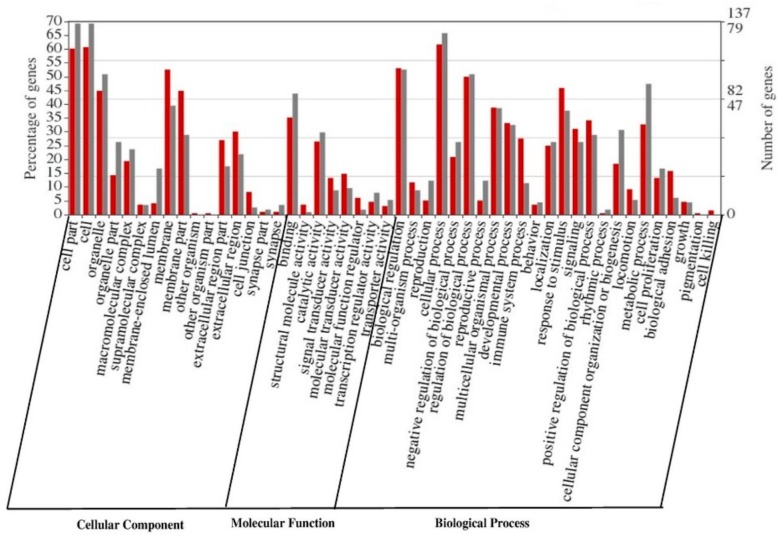
Classification of gene ontology of differentially expressed genes (DEGs). Results are show in three main categories (biological process, cellular component and molecular function) Red (down-regulate) and Gray (up-regulate).

The 354 selected genes were categorized into 48 functional groups of the GO, distributed as follows: 232 genes were categorized in 24 functional groups for biological process (BP); 267 genes in 16 groups for cellular component (CC) category; and 191 genes in 8 groups for molecular function (MF) category. The highest abundance of genes was represented in the CC category (Figure 2).

The enrichment of DEGs in GO terms was tested to gain insights about the biological implications. The GO terms GO: 0009987 (Cellular process) for BP, GO: 0005623 (Cell) for CC, and GO: 0005488 (Binding) for MF, showed high abundance of DEGs into these categories (Figure 2). Regarding to functions associated with reproduction processes, we find that the GO: 0022414 (Reproductive process) and GO: 0000003 (Reproduction) are present in the BP category. The metabolic analysis of the DEGs using KEGG database, classified them into 116 metabolic pathways (Table S2). The top 10 metabolic pathways were identified (Table 4) and only six are related to reproductive processes: calcium-signaling pathway, TGF-β-signal pathway, insulin-signaling pathway, estrogen-signaling pathway, PI3K-Akt-signaling pathway, MAPK-signaling pathway (Table 4). Among these KEGG pathways, were the TGF-β-signaling pathway, estrogen-signaling pathway and PI3K-Akt-signaling pathway. These metabolic pathways are associated with gonadal development, ovarian steroidogenesis, oocyte maturation and steroid hormone biosynthesis [25]. These pathways may be playing an important role in the regulation of follicle development in sheep.

### 3.3. Validation of RNA-seq Data by qRT-PCR

**Figure 3:**
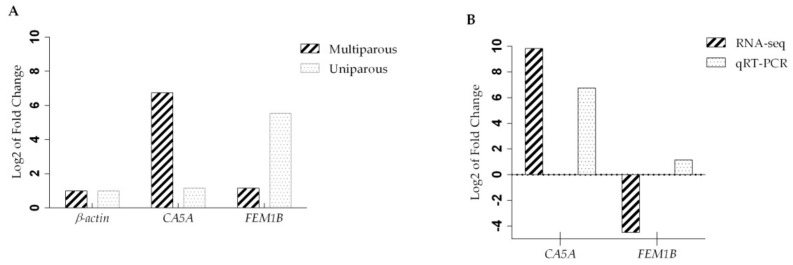
Validation of RNA-seq of *CA5A* and *FEM1B* genes by quantitative real-time polymerase chain reaction (qRT-PCR). (**A**) Gene expression profiles in ovarian tissue in multiparous and uniparous in Pelibuey sheep by qRT-PCR; (**B**) Expression level of the differentially expressed genes detected by the RNA-seq and expression level of the differentially expressed genes obtained by qRT-PCR.

The *CA5A* up-regulated and *FEM1B* down-regulated genes (Table 3), involved in the nitrogen pathway and the regulation of ubiquitin-protein transferase activity, respectively, were selected to confirm their expression profiles obtained by RNA-seq analysis using qRT-PCR analysis. The β-actin gene was used as housekeeping control. The experimental evaluation of the *CA5A* gene using qRT-PCR showed a positive expression, agreed with the results obtained from the RNA-seq analyzes. While in the case of the relative expression of the gene *FEM1B* it was not a negative expression, as was predicted in the RNA-seq analyzes, observing an increase in its expression in the results of qRT-PCR analysis, but its expression being less than in the tissue of the uniparous sheep (Figure 3).

## 4. Discussion

The ovulation rate is determined by the regulation of intraovarian factors and their increase is associated with the expression of genes involved in the development of antral follicles in the preovulatory stage, resulting in an increase in prolificacy traits [8]. The transcriptomic analysis performed with the RNA-seq technique in this study yielded a number of reads similar to other studies (53,024,205) [14] and (86,966,348) [26]. Similarly, with our transcriptomic analysis in the Pelibuey sheep, we have identified genes that are associated with multiple calving sheep and have been previously reported in other sheep breeds such as the *IL1A* (Interleukin 1 α) and *CYP2S1* (Metabolism of xenobiotics by Cytochrome P450) genes in Hu ewes, the function of these genes in the process of folliculogenesis is unknown [14]; the *BMP15* and *INHBA* (Inhibin β A subunit) genes in Finnsheep ewes [26]. The *BMP15* gene has activity in the oocytes (both immature and mature) within the ovary, stimulating the proliferation of GC in a follicle-stimulating hormone receptor (FSHR)-independent manner [2] and *INHBA* gene shows a considered level of mRNA expression in the GC of antral follicles during the stages of the estrous cycle in sheep [27]. While the gene *IL1A* has activity in the mechanism of apoptosis, which is responsible for the elimination of oocytes and a deficiency or blockade of increased expression of FSHR in GC in rodents [26]. The *CYP2S1* gene acts in the synthesis of steroid hormones for the development of ovarian follicles from the preantral stage to the Graafian follicle stage in sheep [24]. These results suggest that genes identified in the present study participate in the development of the final stages of follicles, as well as in the low expression of *IL1A*, which decreases cellular apoptosis in the samples of oocytes.

The analysis of the DEGs, revealed that some of the main genes that are positively regulated are *FGF18, CA5A, SVIP, CXCL14* and *FST*; while, that *RENT, FEM1B, MMP9, ANKRD37* and *CYP2S* are down-regulating as shown in Table 2. The Fibroblast Growth Factor 18 (FGF18) is involved in the signaling of paracrine in the ovarian follicle, differentially activating the receptors depending on the properties of the extracellular matrix in the follicle, suggesting that it activates an apoptotic pathway through FGFR3c (fibroblast growth factor receptor 3) in GC as well as in other cell types [28]. Atresia of the subordinate follicle is also associated with an increase in the expression levels of *FGF18* in theca cells and in the follicular fluid, which in turn acts on GC to inhibit steroidogenesis [29]. Carbonic anhydrase 5A (*CA5A*), is a zinc metalloprotein that catalyze the reversible conversion of CO_2_ to bicarbonate ion and a proton [30]. In previous studies, *CA5A* activity has been reported in cellular functions in mammals, including processes in the ovary [30,31,32]. In these studies, its expression is reported on the surface of the epithelium of the ovary; and is absent from the corpora lutea in humans, pigs, rats, rabbits, mice, cats and dogs, and in the rabbit oocyte [32]. The activity of the *CA5A* gene in granulosa cells has been reported in rats and guinea pigs and is especially outstanding in small developing follicles before the stage of a recognizable antrum [33]. In our study the *CA5A* gene was identified in the zinc ion binding function in the MF of the GO categories. In humans, this gene is involved in the process of ureagenesis, where it supplies HCO_3−_ for carbamoyl phosphate synthetase and in gluconeogenesis for pyruvate carboxylase [33], and the low-expression or deficiency of *CA5A* results in impaired of bicarbonate which predisposes to hyperammonemia and hypoglycemia [34]. Follistatin (*FST*) is identified as member of the TGF-superfamily; the *FST* plays an important role in female physiology by regulating of follicle stimulating hormone (FSH) levels through blocking activin actions and conditional knockout females develop fertility defects, which include reduced litter sizes and, in more severe cases infertility [35]. In this study, the *FST* gene showed a significant expression, which is possibly related to reproductive processes in this breed.

Within the group of down-regulated genes, the Resistin (*RETN*) gene, it is associated with lipid metabolism, cause insulin resistance and decreased adipocyte differentiation [36]. The recombinant resistance (0.1, 1, 10 and 100 ng/mL) in the steroid hormone (i.e., progesterone, androstendione, testosterone, and estradiol) observed in prepubertal sows stimulates the steroidogenesis in the ovarian follicles, increasing the expression of the genes of the cytochrome family (*CYP11A1* and *CYP17A1*) [37]. The Fem-1-Like Death Receptor-Binding Protein α (*FEM1B*) gene intervenes in the insulin secretion and acts on the degradation of Stem-Loop Binding protein (SLBP) [38]. In studies that used mice, an interaction was demonstrated between the products of the *ANKRD37* (Ankyrin Repeat Domain 37) and *FEM1B* genes, which were highly expressed in male germ cells; the binding of the products of the *FEM1B* and *ANKRD37* genes and their interaction resulted in degradation through the ubiquitin-mediated proteolysis pathway [39]. A functional variation (SNP rs10152450 and haplotype GGAAT) in the *FEM1B* gene increases insulin secretion and insulin resistance in mice, and the result is ovarian hyperandrogenism that occurs at the time of puberty in polycystic ovary syndrome (PCOS) [40]. In this study, the *FEM1B* gene showed a negative expression. In the GO functional annotation, the *FEM1B* gene is identified as a transcriptional regulator of ubiquitin-protein transferase activity. Reports by Lim et al. [41] suggest that chronic androgenization is a negative regulator of the growth of the antral follicle and the activity of the androgen receptor mediated by ubiquitin, resulting in antral follicle growth arrest in a chronically androgenized PCOS in rat. The Matrix Metalloproteinase 9 (*MMP9*) gene functions for homeostasis of the extracellular matrix and has a role in LH-induced ovarian steroidogenesis in mouse preovulatory follicles [42]. In Romanov ewes, the activity MMP9 is regulate by the events associated with follicular development, plays a role in the disappearance of P450 aromatase in CG and a progressive decrease in levels of P450 17alpha-hydroxylase in the theca internal, suggesting that MMPs can have a role in atresia of the ovary and is associated with distinct changes in levels of extracellular matrix following hypophysectomy [43]. We identified that the *MMP-9* gene is down-regulated in prolific Pelibuey sheep, and metabolic pathway analyses showed that the *MMP-9* gene have activity in the tumor necrosis factor (TNF)- and estrogen-signaling pathway. The Cytochrome P450, Family 2, Subfamily S, Polypeptide 1 (*CYP2S1*) has a role in the expressed during at all stages of development embryonic in mouse, endogenous substrates have as yet not been determined [44]. In the GO analysis, the *CYP2S1* gene was not annotated, however Luo et al. [14] shown that the CYP2S1 gene was enriched in the metabolism of xenobiotics by cytochrome P450. The Homeobox protein gene HOX-A9 (*HOXA9*), is expressed in the reproductive tract of adult females, allowing further development and may play a role in the establishment and maintenance of pregnancy [45]. In recent human studies, up-expression of *HOXA9* can stimulate the progression of ovarian cancer by promoting an immunosuppressive microenvironment through paracrine effects in peritoneal macrophages [46]. In our study, we observed the *HOXA9* gene being expressed negatively, which may also be associated with the estrus stage.

### Gene Ontology and KEGG Analysis: Metabolic Functions in Folliculogenesis

To investigate the functions of DEGs involved in the reproductive processes of prolific Pelibuey sheep, we performed the GO and metabolic pathway analysis, however only the six most important metabolic pathways are discussed here. Our analysis revealed that six genes identified with negative regulation are found in the calcium (Ca^2+^)-signaling pathway such as *ATP2A3, ERBB3, MYLK4, PLCB2, PTGFR* and *PRKCB*. The Ca^2+^ regulates aspects of cell functions including cell cycle progression, maturation and fertilization of mammalian oocytes [47]. In the TGF-β-signaling pathway the up-regulated genes *INHBA, FST* and *BMP15* were identified; and only the up-regulated gene BMP15 was identified in the ovarian steroidogenesis pathway, which is the metabolism of steroid hormones important for reproductive regulation [14]. The TGF-β-signaling pathway is one of the main metabolic pathways of folliculogenesis in mammals [13]. Previous research showed that *INHBA* functions in follicular maturation and steroidogenesis in Small Tail Han ewes, is associated with increased litter size [13], and *BMP15* is expressed in oocytes regulating the proliferation and differentiation of GC in the final development in sheep [8]. In this study, the *INHBA* and *BMP15* genes were up-regulated. The *INHBA* can act in a negative way by inhibiting the production of FSH, whereas the inhibitory effect of *BMP15* on the action of FSH regulates the development of oocytes. However, a change of nucleotide modifies the protein, increasing the sensitivity to FSH and increasing the rate of ovulation and therefore prolificacy. Insulin signaling pathway, regulates blood glucose levels, maintenance of energy and glucose metabolism, also regulates ovarian androgens producction in humans, which suggests that it has an indirect role in early folliculogenesis, ovarian androgen is important to maintain the development of the follicle [48].

Four genes including *SHC4, HSPA1A, MMP9* and *PLCB2* enriched in the estrogen-signaling pathway were down-regulated. Two up-regulated genes, *FGF18* and *NR4A1*, and seven down-regulated genes (*COL27A1; CSF1R; ITGB7; IL6R; IL7R; SPP1* and *SYK*) were enriched in the PI3K-Akt-signaling pathway.

The *NR4A1* gene was enriched in the pathway of the PI3K-Akt signaling pathway. The *NR4A1* gene plays an important role in the development of the decomposition of the germinal vesicle (GVBD) which is regulated by FSH in cattle [49]. The *IL1A* gene was enriched in the MAPK signaling pathway, where has been reported as an important regulator of the maturation of oocytes in different species [50]. One study by Yu et al. [51] in ovaries of immature rats, suggests that FSH-activated p38 MAPK signal pathway regulates progesterone and estrogen production in GCs differentially.

Six enriched pathways are related to reproductive processes. However, four signaling pathways, the cAMP-signaling pathway, prolactin-signaling pathway, Rap1-signaling pathway and the insulin secretion pathway, have no association with reproductive function in the ovary. However, these pathways were derived from their corresponding physiological processes, we speculate that some of the genes involved in these pathways, may be related to some function of the reproductive processes of the ovary. This could agree with that not all the prolific sheep have point mutations reported as associated to litter size, and there are probably other molecular mechanisms that influence it.

The identifying genes in the Pelibuey sheep, previously reported in other sheep breeds, such as those that regulate the TGF-β-signaling pathway, are a sign that our analyzes are consistent with what was reported by other groups. We assume that the expression of the new genes identified in our study may also be due to other physiological factors, where a marked seasonality is not observed in this sheep and to nutrition, where they are an important factor that regulates reproductive performance of sheep and affects follicle development. Possibly these factors, may be stimulated some of the six enriched pathways found in the ovarian tissue, controlling the litter size. This information serves as a basis to design new experiments to evaluate prolificacy and control these factors in the prolificacy of Pelibuey sheep.

## 5. Conclusions

The analysis of ovarian tissue of sheep that gave birth to twin lambs and sheep that gave birth to a single lamb was performed using the new-generation sequencing technology RNA-seq. The most relevant set of genes identified such as *BMP15*, *INHIB*, *SERPINE5*, *IL1A*, *CD5* and *CA5A* are associated with reproductive processes. In this study we report new genes, not previously documented in sheep such as *ATP2A3*, *MMP9*, *CBL*, *SOCS3*, *FGF18*, *NR4A1*, *CHC4* and *FST* that are involved in the reproduction process. These genes can be considered candidates to regulate prolificacy, because they enriched six pathways with related to function in reproductive processes.

The information generated in this study provides the basis for understanding the reproductive processes in hair sheep such as Pelibuey, as well as providing additional data to aid in future research and breeding programs.

## Figures and Tables

**Table 1:** Primer sequences used to amplify target genes using by quantitative real-time polymerase chain reaction (RT-qPCR). β-actin for the housekeeping as an internal control.

Name	Size (pb)	Primer Sequence	Tm (C°)	Efficiency
*CA5A*	154	F: 5′-CCCCTTGGAAAACCACTACA-3′	55.3	100.0
		R: 5′-TGGTATTTCACAGCGTTCCA-3′		
*FEM1B*	159	F: 5′-AAGGTTTCACCTTGCTGCAT-3′	52.0	106.1
		R: 5’-CATTGTCCACAGCATTCACC-3′		
β-actin	146	F: 5′-TGCGGCATTCACGAAACTAC-3′	55.9	100
		R: 5′-AGGGCAGTGATCTCTTTCTG-3′		

**Table 2:** Total summary of high-quality filtered readings.

File Name	Group M	Group U
Total number of reads	64,998,983	61,941,256
Average length of readings (n)	75.4	75.4
Number total of HQ reads	60,530,690	57,444,313
Percentage of HQ reads (%)	93.60	93.60
Total number of HQ bases in HQ reads	4,364,547,403	4,148,052,967
Number of primer/Adaptor contaminate HQ reads	1,452	1,332
Total number of HQ filtered reads	60,528,631	57,442,547
Percentage of HQ filtered reads (%)	93.11	92.24

HQ: high quality; n: nucleotide.

**Table 3:** The top 10 most significantly affected genes in ewes with two lambs per birth compared with one lambing per birth.

Gene Name	Log2 *FC	*p*-Value	FPKM		FDR	Up/Down-Regulated	Description
			M	U			
*FGF18*	inf	0.00005	0.891434	0	0.000345978522902	Up	Fibroblast Growth Factor 18
*CA5A*	9.82667	0.00005	126.35	0.13914	0.000345978522902	Up	Carbonic anhydrase 5A
*SVIP*	5.61939	0.00005	25.8765	0.526384	0.000345978522902	Up	small VCP interacting protein
*CXCL14*	2.67744	0.00005	23.8323	3.72543	0.000345978522902	Up	C-X-C motif chemokine ligand 14
*FST*	1.32408	0.00005	259.25	103.546	0.000345978522902	Up	Follistatin
*RENT*	–inf	0.00005	6.84467	0	0.000345978522902	Down	Resistin
*FEM1B*	–4.48487	0.00005	557.61	24.9029	0.000345978522902	Down	Fem-1-Like Death Receptor-Binding Protein α
*MMP9*	–3.14351	0.00005	46.1391	5.22128	0.000345978522902	Down	Matrix Metalloproteinase 9
*HOXA9*	–2.26451	5E-05	9.80922	2.0415	0.000345978522902	Down	Homeobox protein *HOX*-A9
*CYP2S1*	–2.93691	0.00005	0.31288	2.39599	0.000664855759107	Down	Cytochrome P450, Family 2, Subfamily S, Polypeptide 1

FC: fold Change; FPKM: reads per kilobase transcriptome per million mapped reads.

**Table 4:** The top 10 pathways of the DEG’s related to the reproductive processes in Pelibuey sheep.

Pathway ID	Number of Genes	KEEG Pathway	Up-Regulate	Down-Regulate
oas04020	6	Calcium-signaling pathway		*ATP2A3*; *ERBB3*; *MYLK4*; *PLCB2*; *PTGFR*; *PRKCB*
oas04350	2	TGF-β-signaling pathway	*FST*; *INHBA*	
oas04910	4	Insulin-signaling pathway	CBL; SOCS3	SHC4; PRKAR1A
oas04151	9	PI3K-Akt-signaling pathway	*FGF18*; *NR4A1*	*COL27A1*; *CSF1R*; *ITGB7*; *IL6R*; *IL7R*; *SPP1*; *SYK*
oas04915	4	Estrogen-signaling pathway		*SHC4*; *HSPA1A*; *MMP9*; *PLCB2*
oas04024	3	cAMP-signaling pathway	*HTR1B*; *FOS*; *HHIP*	
oas04917	3	Prolactin-signaling pathway	*FOS*; *SOCS3*	*SHC4*
oas04010	7	MAPK-signaling pathway	*FOS*; *DUSP4; FGF18*; *NR4A1*	*HSPA1A*; *IL1A*; *PRKCB*
oas04015	10	Rap1-signaling pathway	*FGF18*; *P2RY1*	*FYB*; *CSF1R*; *ITGAL*; *ITGAM*; *ITGB2*; *PLCB2*; *PRKCB*; *SKAP1*
oas04911	2	Insulin secretion		*PRKCB*; *PLCB2*

KEGG: Kyoto Encyclopedia of Genes and Genomes. The complete list of KEGG pathways can be found in the Supplementary Materials.

## Supplementary Materials

The following are available online at https://www.mdpi.com/2073-4425/10/5/358/s1, Table S1: Differentially expressed genes involved in the ovary development process in Pelibuey sheep, Table S2: Metabolic pathways identified and related to reproductive processes in Pelibuey sheep.
